# Psychological Distress and COVID-19 Related Anxiety among Malaysian Women during the COVID-19 Pandemic

**DOI:** 10.3390/ijerph19084590

**Published:** 2022-04-11

**Authors:** Nurul Ilani Abdul Latif, Nor Azlin Mohamed Ismail, Sweet Yi Esther Loh, Abdul Ghani Nur Azurah, Marhani Midin, Shamsul Azhar Shah, Aida Kalok

**Affiliations:** 1 Obstetrics & Gynaecology Department, Faculty of Medicine, Universiti Kebangsaan Malaysia Medical Centre, National University of Malaysia, Kuala Lumpur 56000, Malaysia; ilanilatif9@gmail.com (N.I.A.L.); azlinm@ppukm.ukm.edu.my (N.A.M.I.); lohsy86@gmail.com (S.Y.E.L.); nurazurahag@gmail.com (A.G.N.A.); 2 Psychiatry Department, Faculty of Medicine, Universiti Kebangsaan Malaysia Medical Centre, National University of Malaysia, Kuala Lumpur 56000, Malaysia; marhani@ppukm.ukm.edu.my; 3 Community Health Department, Faculty of Medicine, Universiti Kebangsaan Malaysia Medical Centre, National University of Malaysia, Kuala Lumpur 56000, Malaysia; drsham@ppukm.ukm.edu.my

**Keywords:** COVID-19, psychology, women

## Abstract

The combination of COVID-19 outbreaks and nationwide lockdown led to an increased prevalence of psychological distress among the population, especially women, as they have to cope with greater family and work demands. We aimed to identify the factors contributing to psychological distress among Malaysian women during the COVID-19 pandemic. A cross-sectional study was conducted between October 2020 and April 2021, in a teaching hospital in Kuala Lumpur, Malaysia. A self-administered questionnaire was distributed among women, which consisted of (1) Participant’s demographics, (2) COVID-19 knowledge and awareness, (3) Depression, Anxiety, Stress Scale-21 (DASS-21), and (4) COVID-19-related anxiety. Chi-square test and univariate analysis were performed to determine the significant factors associated with psychological distress. The mean scores for knowledge, COVID-19 anxiety, and DASS-21 subcomponents were compared using the Mann–Whitney *U* test. A total of three hundred and thirty-eight women completed the survey. The majority of respondents demonstrated adequate knowledge (95.6%) on COVID-19. The proportion of our women who reported symptoms of depression, anxiety, and stress were 17.2%, 25.1%, and 0.9%, respectively, resulting in a prevalence of psychological distress of 27.8%. Low education level (*p* = 0.017), unemployment (*p* = 0.028), loss of income (*p* = 0.033), and hospital admission for surgical procedures (*p* = 0.021) were significantly associated with a higher psychological burden. A greater level of COVID-19 anxiety was found among Malays (*p* = 0.027), pregnant women (*p* = 0.013), and those who suffered a loss of income (*p* = 0.038) during this pandemic. The COVID-19 pandemic had a negative effect on women’s psychological wellbeing, especially those from the lower socio-economic background. Therefore, adequate information, as well as support, must be provided to the vulnerable groups during the ongoing pandemic, to lessen their psychological burden.

## 1. Introduction

The global COVID-19 pandemic is caused by the severe acute respiratory syndrome coronavirus 2 (SARS-CoV-2), which was first detected in Wuhan, China. The COVID-19 infection can vary between a mild and severe disease that leads to multi-organ failure and death. Long-term complications have been reported in the disease survivors, ranging from mild symptoms such as chronic cough and shortness of breath to severe complications involving lung fibrosis, heart failure, and ischaemic stroke [1,2]. Obese, elderly, and pregnant individuals, alongside those with chronic illness and immunocompromise, are at high risk of developing severe illness from the COVID-19 infection [3,4].

The Malaysian government had instigated the Movement Control Order (MCO) on 18 March 2020 as an outbreak control measure [5,6]. The nationwide lockdown resulted in the cessation of all socio-economic activities except those considered essential services. Domestic and international travel was halted [5,6] while all educational institutions were ordered to close [5,6,7].

Women are financially affected by the pandemic as they make up over four-fifths of the global workforce [8]. As schools closed and family members became ill, they shouldered the burden of care, which further increased their stress and workloads [9,10,11]. Women are also at risk of contracting the disease as more than 70% of health care sector employees are female [12].

A recent Malaysian survey demonstrated that one in every three people suffered mild-to-severe depression during the nationwide MCO [13]. The predisposing factors to psychological distress include being women, age below forty, pregnancy, children at home, and change in occupational status [14,15,16]. Those with pre-existing psychological conditions are also reported to have worsening symptoms [16,17].

During the pandemic, the majority of hospitals would allocate a lot of resources to deal with COVID-19 patients. This resulted in the rescheduling of clinic appointments as well as elective procedures and surgeries. As the pandemic progressed, more hospitals in the Klang Valley, Malaysia, were designated as full COVID-19 hospitals that only dealt with COVID-19 cases. Our institution remained as a hybrid hospital in which normal health services were still delivered alongside the management of COVID-19 patients [18,19]. A COVID-19 PCR screening was mandated on every patient who required admission or a procedure, to protect the health professionals and reduce the risk of disease transmission.

While universal testing of SARS-CoV-2 was viewed favorably by the majority of health care workers, the impact of negative testing on patients is mixed [20]. Prenatal screening could decrease maternal anxiety about the viral infection to herself, her offspring, and her family [21]. In contrast, asymptomatic pregnant women who lived in the area of a high prevalence of COVID-19 cases reported no change in the pre-existing fear or anxiety, following negative testing [20].

We aimed to assess the psychological distress among women who underwent COVID-19 screening during the pandemic. We hypothesized that pregnant women and those in the younger age group have a high prevalence of anxiety and stress.

## 2. Materials and Methods

### 2.1. Study Design and Participants

This cross-sectional study was conducted between October 2020 and April 2021 in a teaching hospital in Kuala Lumpur. Prior ethical approval was obtained from the Research Ethics Committee of the National University of Malaysia (Research code: JEP-2020-631).

The inclusion criteria were Malaysian women, aged above 18 years old and undergoing COVID-19 screening. Women who reported symptoms of COVID-19 such as fever, cough, sore throat and breathlessness were excluded. Eligible women were given information about the survey and written consent was obtained. A self-administered questionnaire was distributed to the participants using either a google form or a paper-based. Each woman’s socio-demographic and clinical data were recorded.

### 2.2. Instruments

The survey questionnaire was formed based on discussion with a team of experts which consisted of obstetricians and gynaecologists, an epidemiologist, and a psychiatrist.

The questionnaire consisted of three domains that assessed the women’s knowledge and awareness of COVID-19, psychological distress, and COVID-19-related anxiety. The survey was conducted in Malay, the national language of Malaysia.

#### 2.2.1. Knowledge and Awareness on COVID-19 Pandemic

We assessed the women’s knowledge and awareness on the COVID-19 pandemic using a validated 10-item questionnaire which was developed by Mohamad et al. [22]. The response choices were ‘True’, ‘False’, and ‘Unsure’. A correct answer was given 1 point whilst others scored 0 points. The total knowledge score ranged from 0 to 10. Participants’ overall knowledge was categorized into adequate if the score was 50% and above (5–10 points) and inadequate if the score is less than 50% (0–4 points).

#### 2.2.2. Depression, Anxiety, Stress Scale-21 (DASS-21)

DASS-21 is a self-reporting tool measuring characteristic attitudes and symptoms of depression, anxiety, and stress [23]. There are seven items for each emotional state. The Malay version of the questionnaires had been validated among the general Malaysian population [24] with an overall Cronbach’s alpha of 0.90 [25].

The participants were asked to rate the extent to which they have experienced various symptoms over the past week, and the score for each subscale was calculated based on a previous study [26]. The depression scale score was categorised as normal (0–9), mild depression (10–12), moderate depression (13–20), severe depression (21–27), and extremely severe depression (28–42). The anxiety scale score was divided into normal (0–6), mild anxiety (7–9), moderate anxiety (10–14), severe anxiety (15–19), and extremely severe anxiety (20–42). The stress scale scoring was normal (0–10), mild stress (11–18), moderate stress (19–26), severe stress (27–34), and extremely severe stress (35–42).

A respondent who demonstrated symptoms of depression, anxiety, or stress from the calculated score would be considered as experiencing psychological distress.

#### 2.2.3. COVID-19 Related Anxiety

The items in this domain were designed to evaluate anxiety towards COVID-19 infection. Women were asked to give a response to each of the following statements using a scale; 1 (strongly disagree) to 7 (strongly agree).

1. I worry about getting the COVID-19 infection;
2. I worry about my partner getting infected with COVID-19;
3. I worry about my family member getting infected with COVID-19; and
4. I worry about transmitting COVID-19 infection to other people

The total score ranged from 4 to 28. Women who reported higher scores were considered to have a greater level of COVID-19 related anxiety.

### 2.3. Face Validation

Face validation was performed on thirty women who were not included in the final analysis. All women reported that the questionnaire was easy to understand and use.

### 2.4. Statistical Analysis

Wang et al. demonstrated the prevalence of anxiety during the COVID-19 pandemic was 28.8% [27]. Based on the figure, our calculated sample size was 340, taking into account a confidence interval of 95%, margin of error of 5%, and 10% incomplete responses.

The Statistical Package of Social Sciences (SPSS) Version 24.0 (IBM Corp., Armonk, NY, USA) was used to analyse the study data. Data were presented as mean (standard deviation, SD) or number, *n* (percentage, %) for continuous and categorical data respectively.

A Chi-square test was performed to determine the significant factors associated with (1) Depression, (2) Anxiety, (3) Stress and (4) Psychological distress. Multiple logistic regression analysis with adjustment for age and ethnicity was subsequently performed to produce the corresponding adjusted odd ratio (AOR) alongside the 95% confidence interval.

The scores for DASS-21 and COVID-19-related anxiety were inspected for normality using the Kolmogorov–Smirnov test. The internal consistency of the newly designed COVID-19 related anxiety questionnaire was assessed using Cronbach’s alpha. Cronbach’s alpha > 0.7 was regarded as satisfactory. All of the items also underwent exploratory factor analysis to confirm the number of factors. The Kaiser rule (Eigenvalue > 1.0) was applied to determine the number of dimensions to extract, whilst the sampling adequacy was assessed through Bartlett’s test of sphericity and the Kaiser–Meyer–Olkin (KMO).

The DASS-21 subcomponents and COVID-19-related anxiety scores among women with different characteristics were compared. A *p*-value less than 0.05 was considered statistically significant.

## 3. Results

A total 350 women were approached, seven women declined, resulting in a response rate of 98%. We excluded five women due to incomplete responses hence the final number for analysis was 338. Table 1 demonstrates the demographic data of our study cohort. The mean (SD) age of our respondents was 33.4 (7.1) years. The majority of the women (81.4%) were Malays. Around two-thirds of our women received a tertiary level of education and were employed. More than 70% of participants have children aged less than 12 at home but only one-fifth of them live with elderly family members. The majority of our cohort (87.9%) consisted of obstetric patients and just over a quarter of all women had underlying medical problems. Around 16% of women reported a loss of income during the COVID pandemic.

The result for domain knowledge and awareness of the survey is shown in Table 2. The mean (SD) knowledge score for our cohort was 7.63 (1.3). A total of 95.6% of women demonstrated adequate knowledge of COVID-19 illness.

The proportion of our women who reported symptoms of depression, anxiety, and stress were 17.2%, 25.1%, and 0.9%, respectively, as demonstrated in Table 3. Ninety-four women scored positive in at least one category, resulting in a prevalence of psychological distress of 27.8%. Table 3 also demonstrates the associations between women’s characteristics and psychological distress alongside its subcomponents.

Logistic regression analysis revealed that loss of income and surgical procedures were associated with a higher rate of depression among our cohort, while tertiary education demonstrated a negative association with psychological distress. These factors remain as significant predictors following adjustment for age and ethnicity in the subsequent multivariable analysis. Women who experienced a loss of income were almost two and a half times more likely to report symptoms of depression (AOR 2.07, 95% confidence interval, CI 1.03–4.14, *p* = 0.041), while surgery during the pandemic increased the risk of depression by two-fold (AOR 2.09, 95% CI 1.16–3.74, *p* = 0.014). Tertiary education was protective against psychological distress and associated with 44% risk reduction (AOR 0.56, 95% CI 0.34–0.94, *p* = 0.026).

Table 4 depicts the participants’ responses to each item of the COVID-19-related anxiety. The newly designed questionnaire demonstrated good internal consistency with Cronbach’s alpha of 0.931. The Kaiser–Meyer–Olkin (KMO) was 0.843, and Bartlett’s Test of Sphericity reached statistical significance with *p* < 0.001, supporting the sample factorability. Exploratory factor analysis via Varimax rotation confirmed single factor loading. The mean score was 25.64 (4.4). Over four-fifths of women were worried about COVID-19 infection happening to themselves as well as their partners and family members. The majority of them also worried about transmitting COVID-19 to other people.

Table 5 compares the mean scores for knowledge, COVID-19 related anxiety, and the subscales of DASS-21, between different factors. The statistical analysis was conducted via the Mann–Whitney U test as the Kolmogorov–Smirnov test on each domain confirmed a non-normal distribution of data (*p* < 0.001).

Tertiary education (*p* < 0.001) and greater household income (*p* = 0.016) were associated with higher knowledge scores among our women. Women who reported a loss of income during the pandemic scored less in the knowledge domain (*p* = 0.003). Individuals with positive COVID-19 cases among their social contact demonstrated greater knowledge scores than those without (*p* = 0.020).

Malay women demonstrated greater COVID-19-related anxiety compared to the other ethnic groups (*p* = 0.027). Pregnancy was also associated with a higher level of anxiety (*p* = 0.013). Individuals who suffered from loss of income during the MCO demonstrated increased COVID-19-related anxiety (*p* = 0.038), depression (*p* = 0.016), and stress (*p* = 0.033) scores.

Women who underwent surgical procedures during the pandemic scored higher in the depression subscale (*p* = 0.021) while the unemployed reported a greater level of stress (*p* = 0.028).

## 4. Discussion

We present a Malaysian study on women’s knowledge of COVID-19, alongside the psychological distress and disease-related anxiety among them during the pandemic. Our study found that the majority of women had adequate knowledge, with greater scores noted among those of higher socio-economic background and whose social contact had been positive for COVID-19. Non-tertiary education was a risk factor for psychological distress; which had been reported in over a quarter of our cohort. Malay women and pregnant mothers displayed greater levels of COVID-19-related anxiety. Individuals who had a loss of income suffered the most with increased depression, anxiety, and stress.

The prevalence of psychological distress among our women was lower than that of other studies with a similar cohort (41–48.6%) [28,29], and among the general population (53.8–65.1%) [27,30]. Our survey results concur with previous studies which demonstrated that females were more susceptible to developing symptoms of various mental problems during the pandemic [9,10,11,31,32].

Women experienced more psychological suffering, partly because they represent a larger fraction of the workforce that may be adversely affected by the COVID-19 pandemic, such as retail, service industry, and healthcare. [12,16]. Women also played a significant role in their family by providing emotional and financial support. The nationwide lockdown had forced the majority of women to stay at home and resulted in reduced outings, increased home workload, and care for the ill, which contributed to psychological distress [10,11,32].

Various studies demonstrated that poor economic status, lower education level, and unemployment are among positive predictors for depression during the pandemic period [16]. The coronavirus outbreak has resulted in strictly imposed quarantine and a reduction in demands for services and goods [33], which has a negative influence on domestic businesses and industries globally. An exponential rise in the unemployment rates was observed in many countries. Individuals who suffered a financial hardship are at greater risks of developing adverse psychological symptoms due to a reduced quality of life and uncertainty following income loss [34]. A Malaysian study during the COVID-19 pandemic demonstrated that higher income and perceived financial status were associated with better mental health outcomes. Individuals with strong financial situations such as those with adequate savings showed stronger resilience to the financial crisis during the COVID-19 pandemic, hence lesser risk of psychological distress [35].

Our study demonstrated that lower educational level is a significant risk factor for psychological distress; a finding similar to other published studies [36,37]. Individuals’ psychosocial adjustment has been associated with educational level, and during the quarantine period, those with a lower educational level might be more susceptible to the negative financial effect of the imposed lockdown. Those who were less-educated may be also prone to health-related prejudices, which might negatively influence their psychological adjustment during the pandemic [38].

An Iranian study showed that individuals with a COVID-19-positive contact (either among family members, relatives, or friends) reported a higher level of anxiety [39]. This could be explained by a possible increased risk of contracting the disease due to recent contact with the infected person. Concern for the health condition of their social contact who contracted COVID-19 may also contribute to the worry. We found that women with positive COVID-19 cases among their social circle demonstrated a similar trend (though non-statistically significant.). Unsurprisingly, the same group reported higher knowledge scores on COVID-19.

Our study demonstrated that the Malay women displayed a greater level of COVID-19 anxiety. Heightened anxiety towards COVID-19 infection may be associated with increased disease awareness and disease prevention measures. Malay women reported greater knowledge scores than those of other ethnicities, although the results were not statistically significant. A study from neighboring Singapore indicated that pregnant Malay women were more likely to practice safe distancing and frequent hand sanitizing compared to the Chinese [40]. Hand hygiene and other precautionary measures such as wearing a mask and social distancing demonstrated a protective effect against stress, anxiety, and depression symptoms [27,41]. A lesser degree of psychological distress was also observed among the Malays in our study even though the finding did not reach statistical significance.

We also found an increased level of COVID-19 anxiety among pregnant women. Our previously published data demonstrated that over four-fifths of expectant mothers expressed worry about the risk of COVID-19 infection to themselves and their babies [42]. A positive correlation between symptoms of depression and anxiety, and perceived threats of COVID-19 to the life of the mother and baby was confirmed by a recent Canadian survey [43]. The finding is supported by various studies that showed a higher level of psychological distress among pregnant women during the pandemic in comparison to the pre-pandemic cohort [44,45,46].

Our study showed that women who underwent surgical procedures were two times more likely to report symptoms of depression. Previous studies also demonstrated that poor self-rated health and pre-existing medical conditions were associated with greater psychological impact and poorer mental health during the COVID-19 pandemic [32,37,41]. Severe COVID-19 infection among patients with co-morbidities and those with suboptimal health contributes to the elevated anxiety, alongside the fear of developing procedure-related complications and feeling anxious about contracting COVID-19 following hospitalization [41,47]. The majority of surgical procedures performed during the pandemic in our centre were for life-threatening or non-benign cases. The women in our cohort which required surgery were more likely to have other comorbidities including malignancy, which may partly explain our finding. On a separate multivariable analysis, surgical procedures remained as an independent predictor for depressive symptoms even after the adjustment for pre-existing medical condition.

A high level of concern about other family members or children getting COVID-19 infection is associated with greater depression, anxiety, and stress [27,48]. Our data however showed otherwise. Women in our cohort who have children and the elderly in their household reported a lesser degree of psychological burden in all the DASS-21 subscales as well as COVID-19-related anxiety, though the results did not reach statistical significance. This finding is similar to that of the Spanish study which demonstrated lower psychological distress among respondents with children in comparison to without one [32], supporting the evidence that parenthood increases subjective well-being [49].

### Strengths and Limitations

The strengths of this study lie in our good sample size and the newly designed COVID-19-related anxiety survey which demonstrated good internal validity and reliability.

Our study may be limited by its cross-sectional design. The survey was conducted during the second wave of the COVID-19 pandemic [50,51] with relatively lower positive cases and mortalities; which may have contributed to a lesser prevalence of psychological distress and anxiety among our cohort. Therefore, a longitudinal study is essential to ascertain the long-term psychological effect of the pandemic.

The DASS-21 questionnaire is a screening tool which evaluates depression, stress and anxiety symptoms in the past week. As these symptoms may vary depending on the individual’s stressors, our data may not truly represent the true prevalence of psychology distress over a longer period of time. We did not establish any prior diagnosis of psychiatric illness among our cohort, which is also among this study’s limitations.

Our surveyed women came from the urban areas, hence the results of this study may not be generalized to the nationwide population. Women from the Malaysian Klang Valley are more likely to have higher economic status with easy access to the internet and health care as well as greater COVID-19 awareness [22,52].

## 5. Conclusions

The COVID-19 pandemic had a negative effect on women’s psychological wellbeing. Those from the lower socio-economic background are most affected. Knowledge on COVID-19 positively correlated to the level of anxiety with expectant mothers experiencing a high level of anxiety. Therefore, adequate information, as well as support, must be provided to the vulnerable groups during the ongoing pandemic, to lessen their psychological burden.

## Figures and Tables

**Table 1:** Demographics, clinical characteristics, social characteristics.

Maternal Characteristics	*n* (%)
Age, mean (SD)	33.4 (7.1)
≤35	234 (69.2)
>35	104 (30.8)
Ethnicity	
Malay	275 (81.4)
Chinese	36 (10.7)
Indian	15 (4.4)
Others	12 (3.6)
Education	
Primary and Secondary	104 (30.8)
Tertiary	234 (69.2)
Employment	
Employed	232 (68.6)
Unemployed/housewives	106 (31.4)
Household income	
<RM 5000	157 (46.4)
≥RM 5000	181 (53.6)
Loss of income due to COVID	54 (16.0)
Household members	
Children under 12	243 (71.9)
Elderly	75 (22.2)
COVID positive cases	
Family members	22 (6.5)
Friends/neighbors	47 (13.9)
Type of patients	
Obstetric	297 (87.9)
Gynecology	41 (12.1)
Pre-existing medical conditions	
Yes	90 (26.6)
No	248 (73.4)
Procedures	
Vaginal delivery	147 (43.5)
Cesarean section	135 (39.9)
Gynecology surgery	20 (5.9)
Others	36 (10.6)

SD: Standard deviation.

**Table 2:** Knowledge of COVID-19.

Question	Correct Answer *n* (%)
The symptoms of COVID-19 are fever, fatigue, dry cough, body aches	300 (88.8)
Runny nose and sneezing are less common in persons infected with the COVID-19 virus.	112 (33.1)
Currently there is no effective cure for COVID-19	244 (72.2)
Persons with COVID-19 cannot infect the virus to others if they do not have a fever.	283 (83.7)
The COVID-19 virus spreads via respiratory droplets of infected Individuals.	286 (84.6)
The COVID-19 virus is airborne.	67 (19.8)
Wearing face masks can prevent the spread of infection by the COVID-19 virus.	327 (96.7)
To prevent the infection by COVID-19, individuals should avoid going to crowded places and avoid taking public transportation.	324 (95.9)
Isolation and treatment of people who are infected with the COVID-19 virus are effective ways to reduce the spread of the virus.	308 (91.1)
The general isolation period is 14 days	329 (97.3)
Score on knowledge and awareness questionnaire, mean (SD)	7.63 (1.3)
Inadequate (<50%)	15 (4.4)
Adequate (≥50%)	323 (95.6)

**Table 3:** Associations between maternal characteristics and depression, anxiety, stress and psychological distress.

Maternal Characteristics	Depression		Anxiety		Stress		Psychological Distress	
	*n* (%) *	AOR ^#^ (95% CI)	*n* (%) *	AOR ^#^ (95% CI)	*n* (%) *	AOR ^#^ (95% CI)	*n* (%) *	AOR ^#^ (95% CI)
Total	58 (17.2%)	-	85 (25.1%)	-	3 (0.9%)	-	94 (27.8%)	-
Age								
≤35	40 (17.1%)	Ref	55 (23.5%)	Ref	2 (0.9%)	Ref	62 (26.5%)	Ref
>35	18 (17.3%)	0.88 (0.47–1.65)	30 (28.8%)	1.25 (0.74–2.13)	1 (1.0%)	0.97 (0.09–12.03)	32 (30.8%)	1.11 (0.66–1.87)
	*p* = 0.962	*p* = 0.681	*p* = 0.296	*p* = 0.406	*p* = 1.000	*p* = 0.992	*p* = 0.418	*p* = 0.695
Ethnicity								
Non-Malay	15 (23.8%)	Ref	17 (27.0%)	Ref	1 (1.6%)	Ref	20 (31.7%)	Ref
Malay	43 (15.6%)	0.62 (0.32–1.22)	68 (24.7%)	0.96 (0.51–1.80)	2 (0.7%)	0.46 (0.04–5.34)	74 (26.9%)	0.87 (0.47–1.59)
	*p* = 0.121	*p* = 0.170	*p* = 0.710	*p* = 0.895	*p* = 0.463	*p* = 0.532	*p* = 0.440	*p = 0*.642
Education								
Non-tertiary	24 (23.1%)	Ref	31 (29.8%)	Ref	1 (1.0%)	Ref	38 (36.5%)	Ref
Tertiary	34 (14.5%)	0.58 (0.32–1.05)	54 (23.1%)	0.73 (0.43–1.24)	2 (0.9%)	0.97 (0.08–11.46)	56 (23.9%)	0.56 (0.34–0.94)
	*p* = 0.054	*p* = 0.073	*p* = 0.188	*p* = 0.251	*p* = 1.000	*p* = 0.982	*p* = 0.017	*p* = 0.026
Employment								
Yes	36 (15.5%)	Ref	56 (24.1%)	Ref	2 (0.9%)	Ref	59 (25.4%)	Ref
No	22(20.8%)	1.22 (0.66–2.27)	29 (27.4%)	1.07 (0.62–1.85)	1 (0.9%)	1.03 (0.08–13.01)	35 (33.0%)	1.23 (0.73–2.09)
	*p* = 0.236	*p* = 0.530	*p* = 0.527	*p* = 0.808	*p* = 1.000	*p* = 0.983	*p* = 0.149	*p* = 0.441
Household income								
<RM5000	29(18.5%)	Ref	40 (25.5%)	Ref	1 (0.6%)	Ref	45 (28.7%)	Ref
≥RM 5000	29 (16.0%)	1.02 (0.54–1.90)	45(24.9%)	1.08 (0.63–1.86)	2 (1.1%)	1.90 (0.14–26.28)	49 (27.1%)	1.15 (0.68–1.96)
	*p* = 0.551	*p* = 0.958	*p* = 0.896	*p =* 0.775	*p* = 1.000	*p = 0*.632	*p* = 0.745	*p* = 0.596
Loss of income								
Yes	16 (29.6%)	2.07 (1.03–4.14)	16 (29.6%)	1.22 (0.63–2.39)	1 (1.9%)	2.46 (0.20–31.20)	19 (35.2%)	1.31 (0.69- 2.48)
No	42 (14.8%)	Ref	69 (24.3%)	Ref	2 (0.7%)	Ref	75 (26.4%)	Ref
	*p* = 0.008	*p* = 0.041	*p* = 0.408	*p* = 0.551	*p* = 0.408	*p* = 0.486	*p* = 0.187	*p* = 0.416
Pregnancy								
Yes	49 (16.5%)	0.79 (0.34–1.81)	77 (25.9%)	1.70 (0.73–3.94)	3 (1.0%)	-	82 (27.6%)	1.10 (0.52–2.33)
No	9 (22.0%)	Ref	8 (19.5%)	Ref	0 (0.0%)	-	12 (29.3%)	Ref
	*p* = 0.385	*p* = 0.574	*p* = 0.375	*p = 0*.216	*p* = 1.000		*p* = 0.824	*p* = 0.798
Pre-existing medical condition								
Yes	19 (21.1%)	1.41 (0.74–2.66)	23 (25.6%)	0.93 (0.52–1.65)	3 (1.2%)	-	62 (27.1%)	1.22 (0.71–2.11)
No	39 (15.7%)	Ref	62 (25.0%)	Ref	0 (0.0%)	-	32 (29.4%)	Ref
	*p* = 0.246	*p* = 0.295	*p* = 0.917	*p* = 0.798	*p* = 0.568		*p* = 0.661	*p* = 0.475
Surgical Procedure								
Yes	35 (22.6%)	2.09 (1.16–3.74)	46 (29.7%)	1.54 (0.94–2.53)	1 (0.6%)	0.59 (0.05–6.63)	51 (32.9%)	1.60 (0.98–2.59)
No	23 (12.6%)	Ref	39 (21.3%)	Ref	2 (1.1%)	Ref	43 (23.5%)	Ref
	*p* = 0.015	*p* = 0.014	*p* = 0.077	*p* = 0.088	*p* = 1.000	*p* = 0.671	*p* = 0.054	*p* = 0.059
A vulnerable group in the household								
Yes	43 (16.5%)	0.83 (0.43–1.61)	66 (25.4%)	1.02 (0.56–1.84)	3 (1.2%)	-	74 (28.5%)	1.12 (0.63–2.02)
No	15 (19.2%)	Ref	19 (24.4%)	Ref	0 (0.0%)	-	20 (25.6%)	Ref
	*p* = 0.580	*p = 0*.586	*p* = 0.855	*p* = 0.959	*p* = 1.000		*p* = 0.626	*p* = 0.696
Social contact with COVID-19								
Yes	10 (16.7%)	1.20 (0.55–2.62)	17 (28.3%)	1.33 (0.69–2.55)	0 (0.0%)	-	18 (30.0%)	1.36 (0.72–2.58)
No	48 (17.3%)	Ref	68 (24.5%)	Ref	3 (1.1%)	-	76 (27.3%)	Ref
	*p* = 0.911	*p = 0*.643	*p* = 0.531	*p* = 0.390	*p* = 1.000		*p* = 0.676	*p* = 0.344

AOR, adjusted odd ratio; CI, Confidence Interval; Ref, reference; * Chi-square test or Fisher’s Exact where appropriate; ^#^ adjusted for age and ethnicity.

**Table 4:** COVID-19-related anxiety.

Items	Disagree *n* (%)	Neutral *n* (%)	Agree *n* (%)
Worry about COVID-19 infection to:			
Self	4 (1.2)	52 (15.4)	282 (83.4)
Partner	8 (2.4)	39 (11.5)	291 (86.1)
Family member	7 (2.1)	38 (11.2)	293 (86.7)
Worry about transmitting COVID-19 to other people	13 (3.8)	55 (16.3)	270 (79.9)

**Table 5:** Comparisons of mean scores for Knowledge, COVID-19 anxiety, Depression, Anxiety, and Stress between different maternal characteristics.

Women’s Characteristics	Mean (SD)				
	Knowledge Scores	COVID-19 Related Anxiety	Depression	Anxiety	Stress
All, mean (SD)	7.63 (1.3)	25.64 (4.4)	4.01 (6.6)	4.92 (7.0)	5.74 (7.4)
Age					
≤35	7.63 (1.2)	25.65 (4.4)	3.91 (6.3)	4.72 (6.6)	5.42 (7.1)
>35	7.63 (1.4)	25.63 (4.3)	4.23 (7.3)	5.38 (7.9)	6.46 (8.0)
	*p* = 0.909	*p* = 0.749	*p* = 0.983	*p* = 0.853	*p* = 0.351
Ethnicity					
Malay	7.66 (1.2)	25.93 (4.1)	3.85 (6.5)	4.88 (7.1)	5.62 (7.3)
Non-Malay	7.49 (1.5)	24.38 (5.4)	4.70 (7.1)	5.11 (6.7)	6.25 (7.6)
	*p = 0*.716	*p* = 0.027	*p* = 0.253	*p* = 0.669	*p* = 0.443
Education					
Non-tertiary	7.24 (1.3)	25.41 (4.7)	4.33 (6.2)	4.98 (6.4)	6.46 (7.4)
Tertiary	7.80 (1.2)	25.74 (4.2)	3.86 (6.8)	4.90 (7.2)	5.42 (7.3)
	*p* < 0.001	*p* = 0.491	*p* = 0.222	*p* = 0.634	*p* = 0.137
Employment					
Yes	7.69 (1.2)	25.56 (4.5)	3.94 (6.7)	4.71 (7.1)	5.28 (7.2)
No	7.50 (1.4)	25.82 (4.1)	4.15 (6.5)	5.40 (6.7)	6.74 (7.7)
	*p* = 0.202	*p* = 0.726	*p* = 0.573	*p* = 0.067	*p* = 0.028
Household income					
<RM 5000	7.47 (1.3)	25.33 (5.0)	4.00 (6.6)	5.29 (7.1)	6.27 (7.5)
≥ΡΜ 5000	7.77 (1.2)	25.91 (3.8)	4.01 (6.7)	4.61 (6.9)	5.28 (7.3)
	*p* = 0.016	*p* = 0.544	*p* = 0.843	*p* = 0.110	*p* = 0.088
Loss of income					
Yes	7.22 (1.2)	26.83 (2.6)	6.11 (7.7)	6.56 (8.5)	8.30 (9.0)
No	7.71 (1.3)	25.42 (4.6)	3.61 (6.3)	4.61 (6.7)	5.25 (6.9)
	*p* = 0.003	*p* = 0.038	*p* = 0.016	*p* = 0.272	*p* = 0.033
Pregnancy					
Yes	7.65 (1.2)	25.79 (4.3)	3.95 (6.8)	5.03 (7.1)	5.76 (7.4)
No	7.51 (1.6)	24.56 (5.0)	4.44 (5.1)	4.15 (5.8)	5.61 (7.0)
	*p* = 0.880	*p* = 0.013	*p* = 0.137	*p* = 0.800	*p* = 0.907
Preexisting medical condition					
Yes	7.58 (1.3)	25.60 (4.0)	3.90 (6.3)	4.60 (6.5)	5.47 (7.2)
No	7.65 (1.2)	25.73 (5.0)	4.24 (7.3)	5.60 (7.9)	6.31 (7.7)
	*p = 0*.972	*p* = 0.329	*p* = 0.819	*p* = 0.650	*p* = 0.307
Surgical Procedure					
Yes	7.58 (1.3)	25.65 (4.6)	4.97 (7.5)	5.85 (8.0)	6.58 (7.9)
No	7.67 (1.2)	25.63 (4.2)	3.19 (5.7)	4.14 (5.8)	5.03 (6.8)
	*p* = 0.596	*p* = 0.542	*p* = 0.021	*p* = 0.094	*p* = 0.052
Children or Elderly in household					
Yes	7.70 (1.2)	25.62 (4.4)	3.95 (6.9)	4.88 (7.2)	5.68 (7.6)
No	7.40 (1.5)	25.73 (4.2)	4.18 (5.6)	5.08 (6.4)	5.92 (6.6)
	*p* = 0.188	*p* = 0.958	*p* = 0.203	*p* = 0.235	*p* = 0.330
COVID case among social contact					
Yes	7.98 (1.0)	26.15 (4.0)	4.07 (6.2)	4.83 (6.0)	5.07 (6.8)
No	7.55 (1.3)	25.53 (4.4)	3.99 (6.7)	4.94 (7.2)	5.88 (7.5)
	*p* = 0.020	*p* = 0.284	*p* = 0.535	*p* = 0.826	*p* = 0.362

## Data Availability

The data presented in this study are available on request from the corresponding author.
